# O.5.2-8 The development of a curriculum to teach adapted physical activity in elderly as well as patients with chronic diseases in Luxembourg

**DOI:** 10.1093/eurpub/ckad133.249

**Published:** 2023-09-11

**Authors:** Christophe Meyer, Alexis Lion, Robert Mann, Daniel Ley

**Affiliations:** Ecole Nationale de l'Education Physique et des Sports (ENEPS), Ministère des Sports, L-2630 Luxembourg, Luxembourg; Fédération Luxembourgeoise des Associations de Sport de Santé, L-1445 Strassen, Luxembourg; Association Luxembourgeoise des Groupes Sportifs pour Cardiaques, L-1445 Strassen, Luxembourg; Luxembourg Institute of Research in Orthopedics, Sports Medicine and Science, L-1460 Luxembourg, Luxembourg; Division de la médecine préventive, Direction de la Santé, Ministère de la Santé, L-1213, Luxembourg, Luxembourg; christophe.meyer@sp.etat.lu; Ecole Nationale de l'Education Physique et des Sports (ENEPS), Ministère des Sports, L-2630 Luxembourg, Luxembourg

## Abstract

**Purpose:**

The aim of this project is twofold: 1) to design and implement a curriculum to train physical activity (PA) coaches in giving adapted physical activity (APA) sessions for the ageing population as well as for people with chronic diseases, 2) to create a teaching national framework for health enhancing PA, which does not yet exist, to ensure coherence in providing PA sessions depending on the target population.

**Project description:**

Since the 1980s, an offer of APA sessions was implemented progressively by the associations, the communes, and other institutions in The Grand Duchy of Luxembourg for people with chronic diseases as well as for the ageing population. But the hired coaches and activity leaders have different educational backgrounds (physiotherapists, sports scientists etc) due to the lack of a national curriculum. Therefore, the National School of Physical Education and Sports (ENEPS) as a representative of the Ministry of Sports, in collaboration with the Health Directorate (Health Ministry), the “Fédération Luxembourgeoise des Associations de Sport de Santé” and the Ministry of Family Affairs, Integration and the Greater Region is responsible for designing, developing, and implementing a curriculum in APAs as an educational basis. The curriculum is based on the Grand-Ducal regulation and encompasses three specific modules. While the first module focuses on the concept of physical literacy, on general knowledge of human anatomy and physiology as well as soft skills, the second focuses on the physiopathology and on the benefits and recommendations of pursuing a regular PA. The third module is a practical module covering several PAs such as Nordic walking, yoga, Pilates, aquarobics, dance etc. The complete curriculum will encompass approximately 150 hours of teaching and is completed when all modules are validated, i.e. at least 50% of the maximum score, and after a final practical evaluation.

**Conclusions:**

The imminent launch of the curriculum outlines the commitment of the government to promote the importance of health enhancing physical activity at all prevention level, from primary to tertiary. The curriculum supports the development of a pool of future APA coaches that will help to expand the supply of APA training sessions.

